# An efficient coding theory for a dynamic trajectory predicts non-uniform allocation of entorhinal grid cells to modules

**DOI:** 10.1371/journal.pcbi.1005597

**Published:** 2017-06-19

**Authors:** Noga Mosheiff, Haggai Agmon, Avraham Moriel, Yoram Burak

**Affiliations:** 1 Racah Institute of Physics, Hebrew University of Jerusalem, Jerusalem, Israel; 2 Edmond and Lily Safra Center for Brain Sciences, Hebrew University of Jerusalem, Jerusalem, Israel; Norwegian University of Life Sciences, NORWAY

## Abstract

Grid cells in the entorhinal cortex encode the position of an animal in its environment with spatially periodic tuning curves with different periodicities. Recent experiments established that these cells are functionally organized in discrete modules with uniform grid spacing. Here we develop a theory for efficient coding of position, which takes into account the temporal statistics of the animal’s motion. The theory predicts a sharp decrease of module population sizes with grid spacing, in agreement with the trend seen in the experimental data. We identify a simple scheme for readout of the grid cell code by neural circuitry, that can match in accuracy the optimal Bayesian decoder. This readout scheme requires persistence over different timescales, depending on the grid cell module. Thus, we propose that the brain may employ an efficient representation of position which takes advantage of the spatiotemporal statistics of the encoded variable, in similarity to the principles that govern early sensory processing.

## Author summary

Grid cells encode a mammal’s estimate of position by firing in multiple locations. These locations are arranged on the vertices of a triangular lattice. Lattices vary in spacing and therefore represent position over different spatial scales. We suggest that grid cells encode position while taking into account the spatiotemporal statistics of an animal’s movement trajectory. Based on this hypothesis, we develop a theory for efficient encoding of trajectories in the brain. One of the main predictions of our theory is that different numbers of grid cells should be allocated to each spatial scale.

## Introduction

A central goal of systems neuroscience is to unravel the principles of encoding in the brain. It has been conjectured that the neural circuitry in primary sensory areas implements coding schemes that maximize information about sensory inputs, while constraining neural resources such as the number of cells or the rate of spikes. This hypothesis [1] has been particularly successful in explaining neural responses in early visual and auditory areas [2–7]. More recently, it was proposed that grid cells in the entorhinal cortex [8] implement an efficient code for an internally computed quantity, the position of an animal in its environment [9–13]. These cells fire in multiple locations within the animal’s environment, arranged on the vertices of a triangular lattice that tiles the plane. According to the above proposal, the neural code for position, implemented by grid cells, possesses a dynamic range (defined as the ratio between the representable range and the resolution) that depends exponentially on the number of encoding neurons [9–11, 14]. Thus, the dynamic range of the grid cell code vastly exceeds that of unimodal coding schemes, such as the encoding of position by place cells in the hippocampus [15], or the encoding in head direction cells [16].

Previous works that analyzed the grid cell code from a theoretical perspective have drawn extensively on the literature concerned with neural encoding and decoding of static, low-dimensional variables. However, the trajectory of an animal in space is a dynamic variable, which possesses characteristic temporal statistics. Hence, it is interesting to ask whether the structure of the neural code for an animal’s position can take advantage of the temporal statistics of this variable, in analogy with the important role of natural statistics in sensory encoding. We start our analysis of this question with a fairly general discussion on the number of neurons required to encode a dynamic trajectory with a certain precision. When applied to the grid cell representation of position, this argumentation leads to a salient prediction: in an efficient allocation of grid cells to modules, the number of grid cells participating in each module should sharply decrease with the grid spacing (here, the grid spacing is the distance between the periodic firing fields of the grid cell, and a module is defined as a group of grid cells that share the same grid spacing and orientation). We then consider a more detailed theoretical framework that provides quantitative predictions on the distribution of cells across modules, as well as the grid spacings. This theory generates a prediction that population sizes should decay with the grid spacing as a geometric series. In addition, the theory predicts that grid spacings should follow approximately a geometric progression—as predicted previously in theories of static coding [11–13], and in agreement with experimental observations [17].

**Fig 1:**
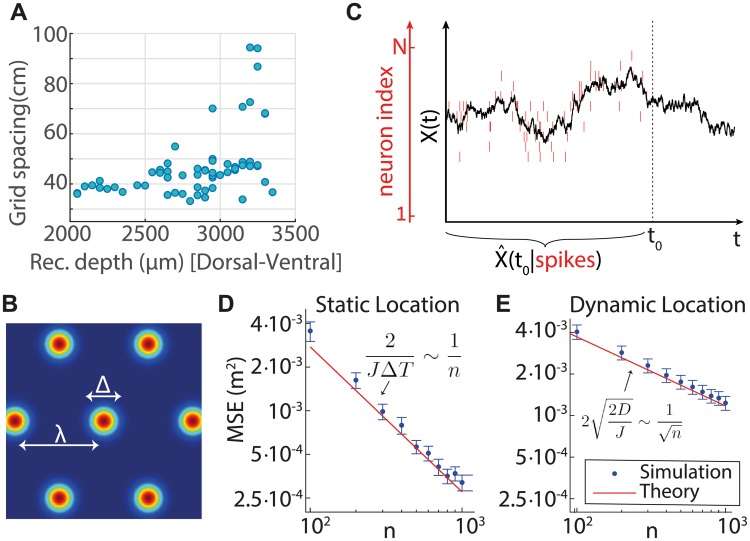
Modular organization and dynamic decoding. **A.** Experimental evidence for the modular organization of grid cells (adapted from [17], Supplementary Figure 2e, Rat 14257): grid spacing in a single rat, where each dot corresponds to a single cell. Note the dramatic decline in the number of cells for larger grid spacings (see also Fig 5H and additional Figs in [17]). **B.** Schematic illustration of the posterior distribution over position, inferred from spikes generated by all cells in a single module. The posterior has the same periodicity λ as the single neuron tuning curves. The local MSE, denoted by Δ^2^, is proportional to the variance of the local probability distribution around each peak. **C.** Schematic illustration of a decoder for a dynamic variable, which follows the statistics of a simple random walk (shown for simplicity in one dimension, and for non-periodic receptive field). Black: a random walk trajectory $\vec{x}\left(t\right)$. Red lines represent spikes emitted by a population of neurons with different tuning curves, where the red *y* -axis represents the neuron index. The decoder estimates the animal’s position at time *t*_0_, based on all the spikes that occurred up to that time. **D.-E.** Local MSEs of an optimal decoder, estimating position based on spikes from a single module, as a function of the number of neurons, for a static two-dimensional variable (D), and a dynamic random variable, following the statistics of a simple random walk in two dimensions (E). Logarithmic scales are used in both panels. Blue dots: measurements of the local MSE from simulations of an optimal decoder, responding to spikes generated by neural populations of varying size. Each dot represents an average over 300 realizations, where in (D) the averaging is over a single readout time interval from each simulation lasting Δ*T* = 100 ms, starting with a uniform prior over positions, and in (E) we average the local MSE in each simulation also over time (realizations lasting at least ∼ 200 ms). Error bars: 1.96 standard deviations of the local MSEs obtained from each simulation, divided by square root of the number of simulations (corresponding to a confidence interval of 95%). The receptive fields of the cells consist of a sum over periodically translated Gaussians with maximal firing rate *r*_max_ = 10Hz and standard deviation *σ* = λ/10, and the grid spacing is λ = 1 m. In the dynamic case (E) *D* = 0.0125 m^2^ /s. Red lines: theoretical predictions from Eqs (1) and (2) (D) and Eqs (2) and (3) (E).

The prediction that grid cell population sizes should vary sharply across modules deviates from the expectation arising from previous works that addressed theoretically the structure of the grid cell code. Most of these works either assumed a uniform distribution of neurons across grid cell modules [9, 11], or deduced that a uniform distribution is expected based on an optimization principle [13, 18]. We note, however, that the trend seen in a recent systematic characterization of grid cell parameters from multiple cells [17] does not appear to support the uniformity of module population sizes: in this study, the number of observed cells decreased sharply with the spacing (an example is shown in Fig 1A, see also Discussion). A recent work [19], which still considers grid cell activity as a code for a static position, predicts a relatively mild and linear variation in the number of cells from one module to the next: across the first four modules (which are the ones observed experimentally), the predicted number of cells decreases at most by a factor of 0.75. Our hypothesis, that the grid cell code is adapted to the dynamic nature of the animal’s trajectory, generates a qualitatively different prediction for the distribution of cells across modules. In particular, we predict a sharp geometric decrease in the number of cells from one module to the next, which may explain the trends seen in Ref. [17].

Our predictions on the distribution of grid cells across modules are derived based on a hypothesis of ideal coding and decoding, without making strong assumptions on readout within the brain. This point of view leaves open an important question, whether neural circuitry in the brain can read out the grid-cell code while taking into account the temporal statistics of the animal’s location. We demonstrate that fairly simple neural circuitry can indeed perform this task. One outcome of this result is an interesting prediction on the processing of spikes downstream of the entorhinal cortex: in neurons that implement a readout of position, the characteristic integration time of incoming spikes is expected to increase monotonically with the grid spacing of the presynaptic grid cell.

Our work provides new theoretical results, in addition to its specific application to grid cells in the entorhinal cortex: on the structure of neural codes that efficiently represent dynamic trajectories, on the principles that govern precise decoding of position from such codes, and on the existence of simple neural mechanisms that can implement accurate readout of dynamic trajectories from neural activity, while taking into account the spatiotemporal statistics of motion.

## Results

### Encoding of a static location

First, we briefly review the theoretical considerations relevant to the representation of a static variable. Imagine an ideal observer that attempts to read out position from the spikes generated by all the neurons in one module with grid spacing λ, over a time interval Δ*T*. If the rate of spikes is sufficiently large, the posterior distribution over position is approximately given by a sum of periodically translated Gaussians (Fig 1B). The spatial periodicity of this distribution is a consequence of the single neuron tuning curves, which all share the same periodic structure. If the individual receptive fields are isotropic and compact, the summed Gaussians in the posterior distribution are individually isotropic as well [18].

Due to the periodicity of the posterior, the representation of position by a single module is ambiguous. This global ambiguity may be resolved by combining the information from different modules [9, 14, 20]. In addition to the global ambiguity, a local ambiguity in the representation arises from the fact that the spiking activity of the encoding neurons is noisy. The local ambiguity can be quantified by defining a local measure of the readout error: we define the local mean square error (*local MSE*) as the mean square displacement between the true position of the animal and the closest peak of the posterior. The local MSE, which we denote by Δ^2^, is also proportional to the mean variance of the Gaussians (Fig 1B). For independent Poisson spikes [21, 22]

$$\begin{array}{c} \Delta^{2}=\frac{2}{J\Delta T}, \end{array}$$ (1)

where the factor 2 comes from the two dimensions and *J*, the Fisher information rate (in each direction in space, see Eq. [S7]) can be written as:

$$\begin{array}{c} J=\alpha \frac{n}{\lambda^{2}}. \end{array}$$ (2)

Here *n* is the number of neurons in the module, and the proportionality constant *α* depends on the detailed shape of the firing fields and on the spiking statistics (see S1 Text section IV for a derivation of *α* for grid cells with Gaussian receptive fields and Poisson spiking statistics). We assumed that neurons within the module cover densely and uniformly all possible phases of the periodic tuning curve, and that the Cramér-Rao bound [23] is saturated. The dependence of *J* on λ can be deduced based on dimensional analysis, relying on the observations that the maximal firing rate is approximately constant in different modules, and that firing fields of grid cells scale in proportion to the grid spacing [8] (therefore, λ is the only spatial length scale characterizing the response in each module). Note that the precision of readout, Eq (1), depends on the choice of the observation time interval, and that the local MSE is inversely proportional to the number of neurons. A numerical demonstration of this relationship is shown in Fig 1D.

Based on Eqs (1) and (2), a uniform allocation of neurons to modules implies that the ratio Δ/λ, the precision of readout relative to the grid spacing, is the same in all modules. Intuitively, this is a plausible requirement, and indeed this relation was postulated (or derived) in previous works: for example, consider a nested coding scheme [11–13], in which the grid spacings follow a geometric series. Let us denote by λ_*i*_ the grid spacings, ordered sequentially (λ_1_ > λ_2_ > …), and by Δ_*i*_ the corresponding precision of readout from each module. Since λ_*i*_/λ_*i*+1_ is the same in all modules, uniformity of Δ_*i*_/λ_*i*_ across modules implies also that the ratio Δ_*i*_/λ_*i*+1_ is uniform across modules. A sufficiently small value of this ratio ensures that readout from each module is accurate enough to avoid ambiguities arising from the periodicity of response in the successive module with smaller spacing. Thus, by choosing a fixed (and sufficiently small) ratio Δ_*i*_/λ_*i*_, it is possible to ensure that ambiguities do not arise in the readout of the code at any scale.

### Encoding of a dynamic location

To see why the dynamic aspect of the trajectory is consequential, let us suppose that the animal’s trajectory follows the statistics of a simple random walk (we relax this assumption later on, in the section *optimization for other trajectory statistics*). We imagine, in addition, that each neuron fires as an inhomogeneous Poisson process with a rate determined by the tuning curve of the neuron, evaluated at the instantaneous position of the animal. Consider an ideal observer, attempting to estimate the animal’s position at time *t*, based on the spike trains from all neurons in a single module, emitted up to that time (Fig 1C). In the S1 Text, Eq. (S8), we show (based on a related calculation [24]) that the local MSE of such an optimal estimator is given by

$$\begin{array}{c} \Delta^{2}=2\sqrt{\frac{2D}{J}} \end{array}$$ (3)

instead of Eq (1), where *D* is the diffusion coefficient of the random walk. A numerical demonstration of this result is shown in Fig 1E. Note that there is no dependence of Δ^2^ on an arbitrarily chosen time interval of readout: due to the random motion there is a limited precision at which the current position can be inferred based on the noisy spikes, even if all past spikes are available to the decoder. Consequently, a certain number of neurons is required in order to encode the trajectory with a prescribed resolution, regardless of an assumption on the time window of observation available to the decoder. A similar conclusion can be reached not only for grid cells with periodic tuning curves, but more generally for the encoding of a random trajectory by a population of neurons with dense, translationally invariant receptive fields.

By plugging Eq (2) in Eq (3), we see that the local MSE is proportional to *n*^−1/2^, instead of the *n*^−1^ dependence of the static case (compare Fig 1D and 1E). This difference in scaling with *n* may seem minor, but using Eqs (2) and (3) we find that in order to achieve a fixed relative precision Δ_*i*_/λ_*i*_ for all modules, it is now necessary to have

$$\begin{array}{c} n∼\frac{1}{\lambda^{2}}. \end{array}$$ (4)

Thus, far fewer neurons are required in modules with large spacing, compared to modules with small spacing. This result can be easily explained in qualitative terms: the relative position of the animal, in relation to the periodic grid response, varies more slowly in the modules with large λ compared to modules with small λ. When decoding spikes from modules with larger λ, an ideal decoder can rely on spikes emitted within a longer period of time in order to estimate the position, relative to the periodicity of the grid. Thus, a smaller number of neurons is sufficient to achieve a desired relative accuracy of readout. The validity of this interpretation is further demonstrated below (*Biological implications for dynamic readout*).

### Optimal module population sizes and spacings

Previous theoretical studies which did not address the dynamic aspect of the trajectory have predicted a geometric progression of the grid spacings [11, 12], with a spacing ratio in successive modules that ranged from 1.44 to 1.65, depending on the detailed assumptions of the theory [13, 25]. These predictions are in qualitative agreement with the experimental measurements [17]. We therefore ask whether our hypothesis, that the grid cell code is adapted to the dynamic nature of the animal’s trajectory, remains compatible with the empirical observations. To do so, we consider how the principles outlined in the previous section influence the allocation of grid cells to modules in a detailed theory in which we optimize, in addition to the number of cells in each module, also the grid spacings.

We consider a nested code [12, 13], and assume that the position is read out sequentially starting from the module with the largest spacing, progressing sequentially to modules with smaller grid spacings. We follow a similar line of argumentation as in [13], but take into account the motion of the animal. Our goal is to minimize Δ_*m*_, the local root mean square error (local RMSE) of readout from the smallest module, while constraining the largest grid spacing λ_1_ and the number of neurons *N* (equivalently, it is possible to minimize the number of neurons while constraining the readout local RMSE). Additionally, we require that ambiguities about position do not arise at any one of the refinement steps. Therefore, we impose a relation between the local RMSE and grid spacing,

$$\begin{array}{c} \Delta_{i}=\beta \lambda_{i+1}. \end{array}$$ (5)

**Fig 2:**
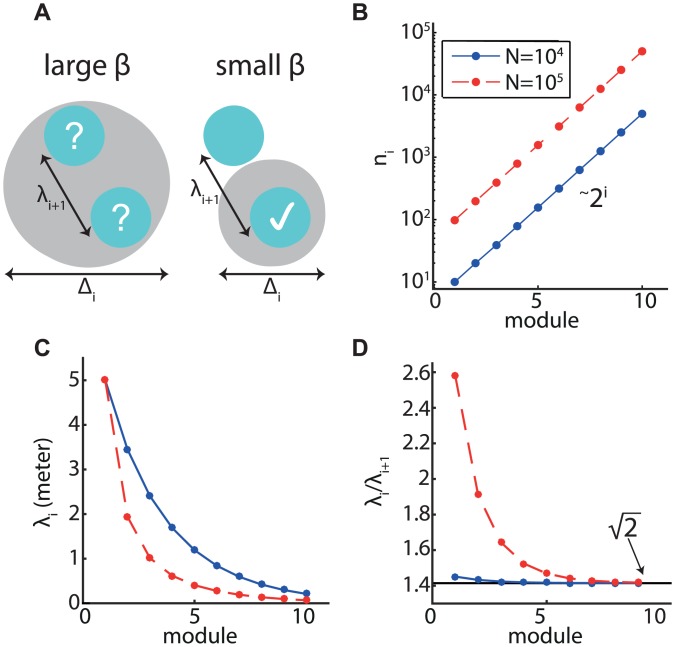
Optimized code: Analytical results. **A.** An illustration of the role of the parameter *β* [Eq (5)]. For large *β* (left), ambiguities in the location readout may arise due overlap of multiple possible locations from the posterior distribution of module *i* + 1 with the posterior distribution based on module *i*. A sufficiently small value of *β* rules out these ambiguities (right). **B.** Number of neurons in a module as a function of the module index (10 modules, ordered by grid spacing starting from the largest spacing). The total number of neurons is *N* = 10^4^ (blue) and *N* = 10^5^ (red). **C.** Grid spacings in the optimized code. **D.** Ratios between grid spacings in successive modules. The ratio approaches $\sqrt{2}$ in the smallest modules. In all three panels, λ_1_ = 5m, *D* = 0.05m^2^ /s, *β* = 0.1, and the receptive fields of the cells are Gaussians with maximal firing rate *r*_max_ = 10Hz.

Here, *β* should be sufficiently small such that the range of likely positions, inferred from module *i*, does not contain multiple periods of the response from module *i* + 1 (Fig 2A). Below, the value of *β* is set as described in the S1 Text section III.A and S3 Fig. Crucially, we use Eq (3) for the local MSE of readout at each step, since we hypothesize that grid cells encode a dynamic position with random walk statistics. Additional details of the optimization are described in the S1 Text section III. The requirement of unambiguous reconstruction [Eq (5)], combined with Eq (3), leads to several salient results. First, we find that in the optimized code, the module population sizes precisely follow a geometric progression:

$$\begin{array}{c} n_{i}∼2^{i}, \end{array}$$ (6)

where *n*_*i*_ is the number of neurons in module *i*, Fig 2B. Second, we find that the ratios between subsequent grid spacings are approximately constant in the modules with small spacing. The optimal ratio approaches a limit given by $\sqrt{2}\;≃\;1.41$ for the smallest modules [Eq (11) and Fig 2D]. This prediction is in close agreement with the ratio of grid spacings in subsequent modules, measured in [17] and averaged across animals, approximately 1.42. Note that the ratios were measured only for the first few modules with lowest grid spacings. Hence, the theory is in very good agreement with the existing measurements, and with previous theoretical predictions that were based on optimal coding of a static variable [13, 25]. For the larger grid spacings, we predict that the ratios may vary monotonically with respect to the spacing (Fig 2D, see S1 Text section III for further discussion). In this respect, our predictions for the spacings deviate to some extent from those of previous works [11–13, 25] that predicted a strictly constant ratio for all modules. Finally, note that Eq (4), obtained from the assumption of a fixed ratio between readout resolution and the grid spacing, is valid for the smaller spacings, as can be seen from Eq (6) and the asymptotic ratio of $\sqrt{2}$ between spacings of successive modules.

The allocated fraction of cells in each module [Eq (6)] is independent of the total number of neurons, the shape of the tuning curve (thus the parameter *α*), the diffusion coefficient *D*, the largest grid spacing λ_1_, the number of modules *m*, and the parameter *β*. Moreover, it remains intact even if we relax the assumption of an optimal estimator, but simply assume the scaling relation Δ^2^ ∼ *J* ^−1/2^, as in Eq (3). The predicted ratios between subsequent grid spacings, for the small spacings, are similarly independent of these parameters as long as the number of modules *m* is sufficiently large. Other, more detailed aspects of the results do depend on parameters. In Fig 2 we assumed that the total number of grid cells is either 10^4^ (blue) or 10^5^ (red), leading to differences in the spacing ratios between subsequent modules—but not in the ratios obtained for the smallest modules (additional examples of how parameters influence the predicted grid spacings are shown in S4 Fig). Most importantly for our discussion on the allocation of grid cells to modules, the module population sizes are given precisely by Eq (6), irrespective of parameters. In particular, about half of the neurons are allocated to the module with the smallest grid spacing (Fig 2B).

**Fig 3:**
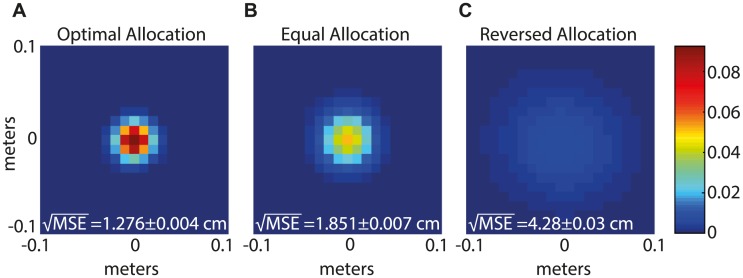
Optimal Bayesian decoder. The posterior probability distribution obtained in simulations of the optimal Bayesian decoder, Eq (12), shifted by the true position of the animal, and averaged over 1350 time points. Three different allocations of neurons to modules are shown: **A.** optimal allocation as in Fig 2B, **B.** equal number of neurons in each module, and **C.** reversed allocation. The MSE and margins of error noted on the left bottom of each panel were computed as in Fig 1D and 1E, based on 100 simulations each lasting ∼ 1.4s. All the parameters are the same as in Fig 2, with *N* = 10^4^.

It may seem surprising that accurate readout is possible at all with only a handful of neurons in the modules with the largest spacing. To test whether this is possible, we characterized the performance of an optimal Bayesian decoder [Eq (12), described in the S1 Text Section I], when applied to simulated spike trains (Fig 3A). The spike trains were generated in response to simulated random walk trajectories, from 10^4^ neurons that were allocated to ten modules based on the optimization scheme discussed above. Accordingly, the module with the largest spacing contained only ten neurons. The root mean square error (RMSE) of the Bayesian estimator is 1.276 ± 0.004 cm. It is instructive to compare this result with the performance under two other allocations of grid cells to modules: if the neurons are allocated with equal proportion to all modules, the RMSE is multiplied by a factor of about 1.5 (Fig 3B). If the allocation of neurons to modules is reversed, such that most neurons participate in the modules with larger grid spacing, the RMSE becomes larger by a factor of about 3.4 (Fig 3C).

In summary, the hypothesis that grid cells are adapted to efficiently encode a dynamic position predicts a sharp decrease in the number of grid cells allocated to modules with large grid spacing, compared to modules with smaller spacing, while remaining compatible with previous theories, which predicted a geometric progression in the grid spacings. A generalization of these ideas to trajectories that do not follow the statistics of a simple random walk is considered later on (optimization for *other trajectory statistics*). First, we consider how the brain might read out the grid cell code while taking into account the animal’s motion.

### Biological implications for dynamic readout

The analysis of grid cell activity from the perspective of an ideal observer is relevant for coding in the brain only if neural circuitry can implement an efficient decoding scheme of the grid cell code, while taking into account the statistics of the animal’s motion. The direct computations involved in a precisely optimal decoder [Eq (12)] are elaborate (see, however, [26]). We next show that in our context it is not necessary to directly implement the optimal Bayesian decoder. A significantly simpler computation, which readily lends itself to neural implementation, can achieve nearly optimal readout of position from each module.

**Fig 4:**
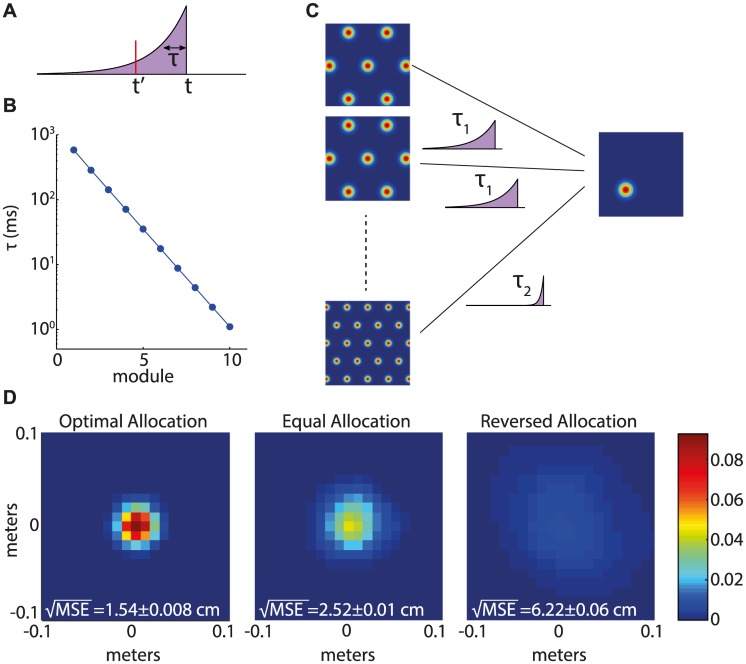
Simplified estimator. **A.** An illustration of the temporal exponential kernel, and the characteristic time *τ*. A spike at time *t*′ contributes to the estimate of position at time *t* with weight $e^{-\frac{t-t^{\prime }}{\tau }}$. **B.** Integration time constant *τ* as a function of the module index, as obtained from Eq (7), substituting the values of λ_*i*_ and *n*_*i*_ from Fig 2 (*N* = 10^4^). **C.** Schematic illustration of a model for readout (e.g. by place cell in the hippocampus). Each place cell approximates the log likelihood to be at a particular position given the spikes of multiple grid cells, as a linear summation of the spikes with integration times that depend on the grid spacing. **D.** Performance of the simplified estimator, measured in the same way as in Fig 3. All the parameters are the same as in Figs 2 and 3, with *N* = 10^4^.

We analyze a simple readout scheme in which spikes emitted by grid cells from a single module are interpreted as if the position of the animal is static. For a truly static position, all the spikes emitted in the past are informative about the current position. Here, however, we consider an estimate of position which is constructed based only on spikes from the recent history, weighted by an exponential kernel with time constant *τ* (Fig 4A). An estimator that treats the position of the animal as if it is static, and attempts to estimate this position based on the recent spikes, has a simple structure: the log likelihood to be at position $\vec{x}$ can be expressed as a linear function of the spike counts [S1 Text, Eq. (S9)]. The estimation of the log likelihood can therefore be implemented by a population of readout neurons: each neuron evaluates the log likelihood to be in a particular position $\vec{x}$. The coefficients appearing in the linear sum can be interpreted as the efficacies of synaptic connections from the grid cells to the readout neurons, whereas the time-dependent kernel can be interpreted as arising from the time course of synaptic currents. Based on the activity of neurons in the readout population, it is possible to identify the most likely position using a simple non-linear operation.

Since the trajectory of the animal is in fact dynamic, the above estimator is, in general, suboptimal. Its best performance is obtained by choosing *τ* as follows [Eq. (S43)],

$$\begin{array}{c} \tau =\frac{1}{\sqrt{2DJ}}=\frac{\lambda }{\sqrt{2D\alpha n}}. \end{array}$$ (7)

This choice balances two contributions to the error of the estimator, with opposing dependencies on *τ* (see also [24]): first, the ambiguity in the decoding of position due to the stochasticity of spikes, which becomes large when *τ* is small (and few spikes contribute to the estimate). The second contribution to the error is due to the animal’s motion. This contribution increases with *τ*, since the simple decoder ignores the animal’s motion altogether. In the S1 Text section II we show that despite its simplicity, the above estimator achieves the same performance as the optimal Bayesian decoder, Eq (3) [see Eq. (S44)], when the readout time *τ* is chosen according to Eq (7).

Based on these results, we next consider a simple model for readout of the grid cell code from multiple modules. For concreteness, we imagine that this readout is performed by place cells in the hippocampus. Each place cell in the readout population approximates the log likelihood to be at a particular position based on spikes generated by grid cells in the entorhinal cortex, Fig 4C. The synaptic activation of the cell is expressed as a linear sum over incoming spikes: the synaptic efficacies are chosen to correctly implement the estimation of the log likelihood [S1 Text Eq. (S10)], and the synaptic current generated in response to each spike decays exponentially with a time constant *τ* that depends on the grid spacing of the presynaptic grid cell (Fig 4C). An exponential nonlinearity is then sufficient to obtain an approximation of the likelihood. Alternatively, lateral inhibitory connectivity in the place cell network, not modeled explicitly here, might implement winner-take-all dynamics [27] which would serve to select a unique estimate for the maximum-likelihood position.

According to Eqs (4) and (7), the time scale *τ* should decrease in sequential modules by a factor of 2 for the modules with smaller grid spacings, where the spacings form an approximately geometric series, and Eq (4) is approximately valid. Characteristic values of *τ* are shown in Fig 4B, where the parameters are the same as in Fig 2. In this example, *τ* varies from ∼1 ms to ∼600 ms, depending on the grid spacing.

With the readout time constants set by Eq (7), and with appropriately chosen synaptic weights, selecting the cell with the maximal activation yields, in response to simulated spikes from 10^4^ grid cells (same as in Fig 3), an estimate for position with a MSE which is close to that of an optimal Bayesian decoder (compare Figs 4D and 3). Thus, a simple neural circuit can implement near-optimal readout of the dynamic trajectory.

An interesting prediction follows for the readout of position in the hippocampus (or in other brain areas), based on inputs from grid cells: Spikes in grid cells are expected to influence the activity of a postsynaptic readout cell over an integration time that depends on the grid spacing of the presynaptic grid cell. The integration time, Eq (7), should increase monotonically with grid spacing (Fig 4B).

### Optimization for other trajectory statistics

So far we considered motion that follows the statistics of a simple random walk. We have done so because the simple random walk is an elementary form of random motion, which is easily amenable to analytical investigation. However, within the above simple readout scheme, it is possible to adjust the grid spacings and module population sizes in order to optimize the resolution of readout for trajectories that are characterized by other statistics. Let us suppose that the mean square displacement of the animal over a time interval Δ*T* follows a power law:

$$\begin{array}{c} \left\langle {\left|\Delta \vec{x}\right|}^{2}\right\rangle =g{\left(\Delta t\right)}^{\epsilon }, \end{array}$$ (8)

where the prefactor *g* and the exponent *ϵ* are constants. An exponent *ϵ* = 1 characterizes a simple random walk, whereas an exponent *ϵ* = 2 characterizes motion at a constant velocity. It is straightforward to evaluate the readout error of the simple estimator in each module under this type of motion (see S1 Text section III.B), and to find the value of *τ* that minimizes its MSE, Eq (17). The optimal MSE, Eq. (S59), scales as *J* ^−*ϵ*/(*ϵ*+1)^, generalizing our result for *ϵ* = 1 [Eq (3)].

We thus repeat our optimization scheme for the number of cells in each module and the grid spacings, while using Eq. (S59) instead of Eq (3). We find that for all plausible values of *ϵ* the qualitative conclusions are the same as those obtained for simple random walk statistics: module population sizes decrease sharply with grid spacing, precisely following a geometric series, and the ratios of successive grid spacings are approximately constant for the modules with small spacing. The predicted ratio between the number of grid cells in successive modules is now given by

$$\begin{array}{c} \frac{n_{i+1}}{n_{i}}=\frac{\epsilon +1}{\epsilon }, \end{array}$$ (9)

and the asymptotic ratio between grid spacings is given for large *i* (small spacings) by

$$\begin{array}{c} \frac{\lambda_{i}}{\lambda_{i+1}}\to {\left(\frac{\epsilon +1}{\epsilon }\right)}^{\epsilon /2} \end{array}$$ (10)

**Fig 5:**
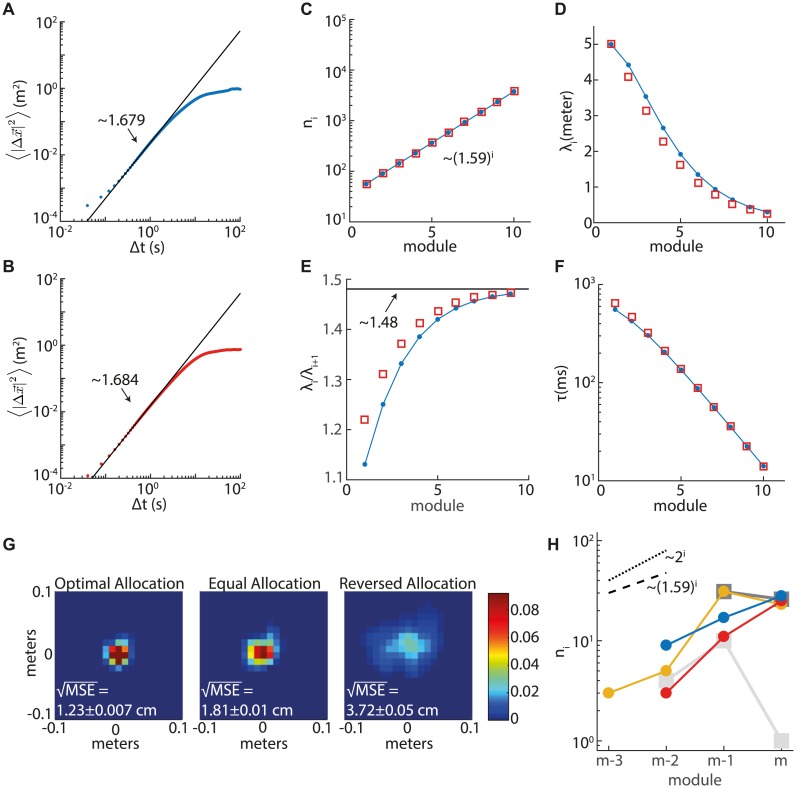
Optimization for rat foraging statistics. **A.-B.** Analysis of trajectories measured from two rats (each panel represents one animal). Both animals randomly foraged for food pellets in a familiar 1.5m square arena with black walls and floor (data courtesy of the Moser lab, NTNU, Trondheim). We evaluated the mean square displacement of an animal $\langle {\left|\Delta \vec{x}\right|}^{2}\rangle$ over a time interval Δ*T*. A fit to a power law [see Eq (8)] was obtained by linear regression of $\text{log }\langle {\left|\Delta \vec{x}\right|}^{2}\rangle$ vs. log Δ*T* in the interval Δ*T* = [0.32, 0.8] s (see the main text for a discussion on the behavior for large and very small Δ*T*). For both animals we obtained *ϵ* ≃ 1.68.**C.-F.** Optimized parameters for encoding and decoding by grid cells, as in Figs 2 and 4B, but using the optimization for power law statistics of motion, and substituting the parameters *ϵ* and *g* [see Eq (8)] that were computed from animal 1 (**A**) (blue dots) and from animal 2 (**B**) (red squares). **C.** Number of neurons in a module as a function of the module index. **D.** The grid spacing. **E.** Ratio between grid spacings in subsequent modules. This ratio approaches ∼ 1.48 in the smallest modules. **F.** Integration time *τ* as a function of the module index, Eq (17). See main text for parameters. **G.** Performance of the simple estimator, using the optimization for power law statistics of motion and substituting the results of C-F. Here the average of the posterior probability distribution is over 9350 time points. The MSE and margins of errors were computed based on 100 simulations each lasting ∼ 9.4s.**H.** Numbers of experimentally identified neurons in different modules as a function of the module index. Data is extracted from Supplementary Figs. 2a and 2e of [17] (see also Fig. 1d in [17] which includes a subset of the data). The numbers are shown for all the tangential recordings. Different colors correspond to different rats (yellow—14257, red—15444, blue—13473, dark gray—13388, light gray—14760). The light and dark gray traces with square symbols correspond to animals in which the coverage of the dorsoventral axis was highly nonuniform (see middle panels of Supplementary Fig. 2a [17]). In rat 14760 (light gray) the number of recorded cells was also significantly smaller than in the other animals. Black lines: predicted slopes for trajectories with random walk statistics [Eq (6),dotted line], and for the power law statistics of measured rat trajectories (∼ 1.59^*i*^, dashed line).

For *ϵ* = 1 these expressions reduce to our previous results for a simple random walk, whereas for *ϵ* = 2 we obtain a predicted ratio of 1.5 between the population sizes in successive modules (instead of 2 for a simple random walk) and an asymptotic ratio of 1.5 between the grid spacings of successive modules (instead of 1.41). We next tested whether Eq (8) is relevant to the motion of rodents by analyzing the trajectories of two rats, which were recorded while the animals were foraging for randomly placed food pellets in a featureless 1.5 m square arena. The two rats exhibit remarkably similar mean square displacements, measured as a function of the time interval (Fig 5A and 5B). Over time intervals ranging from ∼0.1 s to several seconds the mean square displacement is very well fit by a power law with an exponent *ϵ* ≃ 1.68. It is noteworthy that studies on trajectories of other foraging animals have reported on power laws with similar exponents, ranging from 1.6 to 1.7 [28–30]. Over time intervals longer than ∼10 s the mean square displacement saturates due to the finite size of the environment, and on very short time scales (up to ∼100 ms) we observe extra motion with a small amplitude (up to a few centimeters), which is likely an artifact arising from motion of the head.

Assuming an exponent *ϵ* = 1.68 we predict a ratio of approximately 1.6 between the number of cells in successive modules, and an asymptotic spacing ratio of approximately 1.48. These predictions do not dependent on model parameters.

As in the case of random walk statistics, detailed predictions for the grid spacing do depend on parameters, such as the total number of grid cells *N*, the number of modules *m*, the largest grid spacing λ_1_, the prefactor *g*, and the parameter *β*. Fig 5 shows results for *N* = 10^4^, *m* = 10, λ_1_ = 5 m, *g* ≃ 0.023 m^2^/s*^ϵ^* (blue) or *g* ≃ 0.015 m^2^/s*^ϵ^* (red) (extracted from the trajectories), and *β* = 0.03, set to obtain a smallest grid spacing λ_*m*_ ≃ 25 cm. Qualitatively, all the conclusions obtained for simple random walk statistics remain valid for the empirical statistics observed in Fig 5A and 5B.

In Fig 5G we demonstrate the performance of the simplified decoder in response to simulated spikes of 10^4^ grid cells, using the experimentally measured trajectories. The grid spacings and the allocation of the cells to modules follow the optimization results shown in Fig 5C and 5D. As shown in Fig 4D for random walk trajectories, the MSE is significantly smaller in the case of optimal allocation of neurons to modules, in comparison to equal allocation or the reversed allocation.

Finally, Fig 5H shows, using a semilogarithmic scale, the slopes in the dependence of *n*_*i*_ on the module index, as predicted theoretically for the empirical statistics of recorded rat trajectories (dashed line) and for random walk trajectories (dotted line). These are compared with cell counts from several animals, taken from Ref. [17] (Figure 1d, and Supplementary Figure 2a,e). The experimental cell counts exhibit a clear tendency to decrease with the module spacing, even though this dependency is not always monotonic (*e.g.*, in the yellow trace). The overall behavior is qualitatively similar to the prediction of the theory, over a wide range of assumptions on the statistics of motion (see also Discussion).

## Discussion

In summary, we explore a hypothesis that the representation of position in the entorhinal cortex takes advantage of the continuous temporal statistics of motion in order to efficiently encode the animal’s position. This is possible due to the multiscale structure of the grid cell code: in modules with larger grid spacing, the encoded variable varies less rapidly than in modules with smaller spacing. Spikes from these modules remain informative about the current position over a longer time scale, allowing for an efficient encoder to allocate a smaller number of cells to these modules.

From the theoretical perspective, it is interesting to consider the dynamical range of the representation: the ratio between the represented range and a measure of the resolution such as the RMSE [14]. It is known that this ratio can be exponentially large in the number of modules [9–12], but the combination of grid spacings and the cell distribution across modules that optimize this quantity are not known, even when assuming that the encoded variable is static. Our goal here was not to fully solve this problem, but to explore the salient consequences, arising from a hypothesis that the code is adapted to the dynamics of the animal’s trajectory. Therefore, we focused our analysis on nested codes, and assumed that the range of representation of the grid cell code matches the largest grid spacing. However, the relationship between the number of cells in a module and the precision at which the module as a whole encodes a dynamic position is very general. Therefore, we expect that the principles revealed here for the allocation of cells to modules apply also if the range of positions encoded by grid cells is much larger than the largest spacing [9, 10].

The most important prediction arising from our hypothesis, is the highly non-uniform distribution of grid cells across modules. In a recent characterization of grid parameters of multiple cells from several animals, spread across the dorsoventral axis [17], many more cells were found in modules with small spacing compared to modules with larger spacing (Figs 1A and 5H). The study by [17] did not attempt to quantify precisely the population sizes in different modules, and the reported numbers were likely influenced to some extent by experimental biases. In particular, a nonuniform coverage of the dorsoventral axis may lead to such biases: indeed, cell counts from two animals in which the coverage was highly non-uniform (Fig 5H, gray traces) deviate from the trends seen in the three other animals. In addition, the fairly small cell numbers imply a significant statistical error when attempting to infer the cell distribution from the data. Overall, however, the trend observed in [17] is sufficiently pronounced to suggest a non-uniform distribution of grid cells across modules. Additional experiments will be necessary in order to establish this conclusion more firmly, and to obtain quantitative estimates of the distribution, which could be compared with our detailed quantitative predictions.

We predict that module population sizes should follow a geometric series. This conclusion is quite robust, since it arises for any power law dependence of the mean square distance traveled, measured as a function of time. The predicted ratios between the number of cells in successive modules range between 1.5 and 2 for reasonable values of the exponent *ϵ*. The ratio is approximately equal to 1.6 for *ϵ* ≃ 1.7, the value of *ϵ* which we observe in trajectories of randomly foraging rats.

In addition to the probing of multiple cells using movable electrodes as in [17], other emerging techniques may assist in probing the functional properties of hundreds or thousands of cells from a single animal, thus providing more accurate data on the relative numbers of grid cells in different modules: these may include neural recordings using high density silicone probes, and calcium imaging in head-fixed [31, 32] or freely behaving animals [33]. Calcium imaging, in particular, has the advantage that it may generate less bias in the selection of cells for analysis in comparison to electrode recordings. In addition, wireless recordings in large environments or recordings in virtual environments may help assess whether the MEC includes more than 3-4 modules (as observed in [17]), and on the frequency of grid cells from larger-scale modules if they exist. Finally, if future experiments will show that grid cells from the same module are anatomically clustered [34], this may help in estimating the number of cells in each module by sampling representative cells from each anatomical cluster.

The total number of grid cell modules in the rodent brain is not known, and grid spacings have been measured only for the 3-4 modules with the smallest spacing [17]. The observed ratio of ∼ 1.4 between successive spacings [17] is compatible with the predictions of our theory only if the total number of modules is sufficiently large (≳ 6, S4I Fig). In addition, the theory may provide an upper bound on the number of modules: if module population sizes follow a geometric series as we predict, the number of modules is limited by the fact that the largest module must contain at least one neuron. For example, if the total number of grid cells is 10^5^ and the ratio of succesive population sizes is 2, then the number of modules cannot exceed 16. It is important to keep in mind, however, that mechanistic constraints which are not taken into account in our theory may limit the sharp decrease in the population sizes and prevent them from decreasing below a certain size: for example, it may be necessary to include a certain number of cells in each module in order to maintain a continuous attractor [35]. Even in this case, we expect the population sizes to sharply vary in the modules with smaller spacings, which contain a relatively large number of cells. Finally, we note that if grid cell population sizes follow the predictions of our theory, it may be difficult to identify cells with large spacing even in very large environments, due to their scarcity.

Another intriguing prediction of our theory arises from the identification of a relatively simple decoding scheme that takes into account the dynamic aspect of motion: action potentials of grid cells are expected to affect the activity of postsynaptic readout cells over varying time scales, which increase with the grid spacing of the presynaptic cell. A direct test of this prediction would require simultaneous intracellular recording from a postsynaptic readout cell (possibly in the hippocampus) and stimulation of presynaptic grid cells with identified functional characteristics.

According to our theoretical results, predicted time scales should span about three orders of magnitude, from ∼1ms to ∼1s, assuming that the largest grid spacing is ∼5m. Integration time scales up to ∼100ms can clearly be implemented in neural circuitry by the dynamics of synaptic integration. Longer time scales of integration in the order of 1s may require other mechanisms for persistence (reviewed in [36]): these can potentially rely on recurrent connectivity, on short term synaptic plasticity [37] or perhaps on intrinsic cellular persistence. It is noteworthy that intrinsic persistence, with characteristic time scales of seconds has been widely observed in the hippocampal formation, and specifically in the entorhinal cortex [38] and the hippocampus [39].

Our model for readout of grid cell activity, possibly by place cells, was deliberately simplified in order to emphasize the main principles governing the readout of a dynamic variable. Thus, we described the readout as occurring in a single feedforward layer. We speculate that the functional organization along the dorsoventral axis of the hippocampus may be helpful in implementing different time scales for integration at different spatial scales. Furthermore, several lines of experimental evidence suggest that place cells are driven by environmental sensory inputs that are independent of grid cell activity [40, 41]. Nevertheless, there is also compelling evidence that grid cells contribute to the activity of place cells, perhaps most prominently when direct sensory cues are absent, and that the medial entorhinal cortex and hippocampus form together a processing loop responsible jointly for spatial representation, computation, and memory [40, 41] (see, also, [10] for a discussion of possible implications from a theoretical perspective).

In line with the conclusions of previous theoretical studies, we estimate that the grid cell code can represent position with very high precision, in the order of one centimeter (Figs 3–5). This high precision may be required to avoid accumulation of errors over time if the entorhinal cortex is involved in the update of the representation based on idiothetic motion [8, 35, 42], and in the maintenance of short-term memory. In short term memory networks, the fidelity of the neural code is consequential for self-maintenance of the persistent state, in addition to its significance for downstream readout, since memory networks must “read out” their own spikes in order to maintain their persistent state [24]. Thus, we speculate that the efficiency of the grid cell code is beneficial not only for downstream readout of grid cell activity, but also for the stability of the representation within the entorhinal cortex, and that recurrent connectivity within the entorhinal cortex may follow principles of dynamic readout similar to those proposed here explicitly for readout in the hippocampus.

## Methods

### Optimized code

Details of the optimization are provided in the Supplemental Information (Section III). The ratio λ_*i*_/λ_*i*+1_ (Fig 2D) is given by:

$$\begin{array}{c} \frac{\lambda_{i}}{\lambda_{i+1}}=\sqrt{2}\cdot {\left(\lambda_{1}\sqrt{\frac{\alpha N\beta^{4}}{8D\left(\text{1-}{\left(\frac{1}{2}\right)}^{m}\right)}}{\left(\frac{1}{2}\right)}^{\frac{m+2}{2}}\right)}^{{\left(\frac{1}{2}\right)}^{i}}, \end{array}$$ (11)

For simplicity, we assume Gaussian receptive fields, for which the factor $\alpha =4\pi r_{\text{max}}/\sqrt{3}$ in Eq (2), where *r*_max_ is the maximal firing rate (see Supplemental Information, section IV).

### Optimal Bayesian decoder

The posterior probability distribution used by the optimal Bayesian decoder (Fig 3) is obtained using the dynamic update rule:

$$\begin{array}{c} p\left(\vec{x};t+dt\right)=\frac{1}{Z}\left[\int d{\vec{x}}^{\prime }p_{D}\left(\vec{x}|{\vec{x}}^{\prime }\right)p\left({\vec{x}}^{\prime };t\right)\right]p_{\text{spikes}}\left(\vec{x};t\right), \end{array}$$ (12)

where $p_{D}(\vec{x}|{\vec{x}}^{\prime })$ is the probability for the random walk to reach $\vec{x}$ at time *t* + *dt* from position ${\vec{x}}^{\prime }$ at time *t*, and $p_{\text{spikes}}\left(\vec{x};t\right)$ represents the likelihood of the spikes observed within the short time interval, given the position $\vec{x}$ (see S1 Text section I, for more details). The optimal Bayesian decoder estimates the location of the animal by:

$$\begin{array}{c} {\hat{\vec{x}}}_{\text{ML}}={\text{argmax}}_{\vec{x}}p\left(\vec{x};t\right). \end{array}$$ (13)

### Exponential kernel decoder

The posterior probability distribution of the temporal exponential decoder, illustrated in Fig 4D is given by:

$$\begin{array}{c} p\left(\vec{x};t\right)=\frac{1}{Z}\text{exp}\left\{\sum_{i}\sum_{\mu \in i}\text{ln}\left[f_{i}\left(\vec{x}-{\vec{x}}_{\mu }\right)\right]\int_{-\infty }^{t}dt^{\prime }h\left(t-t^{\prime }\right)\xi_{\mu }\left(t^{\prime }\right)\right\}, \end{array}$$ (14)

where the second sum is over neurons *μ* that belong to module *i*, *f*_*i*_(*x*) is the shape of the tuning curve, characterizing the receptive field of the neurons in the *i*th module, ${\vec{x}}_{\mu }$ is the center of the receptive field of neuron *μ*, *ξ*_*μ*_(*t*) is a series of delta functions that represents the spikes of neuron *μ*, *Z* is a normalization constant, and *h*_*i*_(*t*) is the temporal kernel of module *i*. In our case:

$$\begin{array}{c} h_{i}\left(t\right)=\text{exp}\left(-t/\tau_{i}\right) \end{array}$$ (15)

This decoder estimates the location of the animal as in Eq (13), but using the posterior probability distribution from Eq (14).

### Power law statistics of motion

In this case the ratio λ_*i*_/λ_*i*+1_ (Fig 5E) is given by

$$\begin{array}{c} \frac{\lambda_{i}}{\lambda_{i+1}}={\left(\frac{\epsilon +1}{\epsilon }\right)}^{\frac{\epsilon }{2}}{\left[\lambda_{1}^{\frac{1}{\epsilon }}\beta^{\frac{\epsilon +1}{\epsilon }}\sqrt{\frac{\alpha N}{M^{\frac{\epsilon +1}{\epsilon }}}\frac{\frac{1}{\epsilon +1}{\left(\frac{\epsilon }{\epsilon +1}\right)}^{\left(m+\epsilon \right)}}{1-{\left(\frac{\epsilon }{\epsilon +1}\right)}^{m}}}\right]}^{{\left(\frac{\epsilon }{\epsilon +1}\right)}^{i}}, \end{array}$$ (16)

and the optimal time constant for readout *τ* is given by

$$\begin{array}{c} \tau ={\left(\frac{2}{Jg\epsilon \Gamma \left(\epsilon +1\right)}\right)}^{\frac{1}{\epsilon +1}}, \end{array}$$ (17)

where Γ is the Gamma function.

### Spiking simulations

In Figs 1D and 1E, 3 and 4D, trajectories were randomly generated using simple random walk statistics. In Fig 5 we used recorded rat trajectories, as described in the Figure caption. Receptive fields in all simulations were sums of periodically translated Gaussians, with maximal firing rate *r*_max_ = 10 Hz and standard deviation *σ* = λ/10. Grid spacings and allocations of cells to modules were set as described in the text. The orientation of each module, and the spatial phase of each cell, were chosen randomly. Each neuron emitted a spike train with inhomogeneous Poisson statistics, with a time dependent rate determined from the receptive field of the neuron, evaluated at the instantaneous position of the animal. In the optimal Bayesian decoder (Fig 3) the estimate of position was evaluated using Eqs (12) and (13). In the kernel (or simplified) decoder (Figs 4D and 5G) the estimate of position was obtained using Eq (13), where $p(\vec{x};t)$ was evaluated using Eqs (14) and (15) and *τ*_*i*_ were set according to Eq (7).

## Supporting information

S1 Text (PDF)

S1 Fig Local MSE of a kernel decoder with a rectangular kernel. The local MSE for estimate of position from spikes in a single module, using the rectangular kernel. The blue dots are simulations of the MSE’s where each dot is an average over 200 realizations and for each realization the MSE was computed by averaging over time. The margins of error are 1.96 standard deviations of the MSEs divided by the square root of the number of realizations. For each realization we simulate 600 neurons with Gaussian receptive fields where *r*_max_ = 10Hz, and λ = 1m. The red line is the theoretical prediction from Eq. (S39).
(EPS)

S2 Fig Local MSE of a kernel decoder with an exponential kernel. The MSE of the kernel estimator based on spikes from a single module, using an exponential kernel, shown as a function of the integration time *τ*. The blue dots are evaluations of the MSE from simulations, and the red line is the theoretical prediction, Eq. (S42). The parameters are the same as in S1 Fig. Note that the minimum of *V*_exp_ is lower that the minimum of *V*_rect_ (S1 Fig).
(EPS)

S3 Fig Dependence of the MSE on parameter *β*. MSE of the exponential kernel decoder, estimating position based on spikes from all modules, for different values of the parameter *β*. The blue dots are simulations of the MSE’s where each dot represents an average over time and over 20 realizations, each lasting ∼ 700ms. The margins of error are 1.96 standard deviations of the MSEs divided by square root of the number of realizations. The parameters are the same as in Fig 2 with *N* = 10^4^. It can be seen that the MSE has a minimum for some 0.1 < *β* < 0.2. The sharp increase in the MSE for larger values of *β* is the result of ambiguities in the inference of the animal’s position, which occasionally result in a very large displacement between the estimate and the true position. When *β* is larger than the minimum but close to it, these events occur rarely, but generate a large contribution to the MSE. The large error bars are due to the sparseness of these events.
(EPS)

S4 Fig Results of optimization for different choices of the largest grid spacing, the number of modules and the total number of cells. **A.-C.** Analytical results as shown in Fig 2B–2D, using the same parameters as in Fig 2 for *N* = 10^4^ except for a change in one parameter: λ_1_ = 20m (blue) or the number of modules *m* = 7 (red). The detailed spacings depend on the specific parameters. However, the central properties discussed in the manuscript are unchanged (the constant ratio between module population sizes [Eq (6)], and the approximately constant ratio between subsequent grid spacings, for small spacings. **D.-F.** The smallest grid spacing λ_*m*_ as a function of several parameters. **G.-I.** λ_*m*_/λ_*m*−1_ as a function of the same parameters. The results are fairly insensitive to changes in λ_1_ (**D,G**) or in *m*, for sufficiently large *m* (**F,I**). Changing the total number of cells does affect the smallest grid spacing λ_*m*_ (**E**).
(EPS)
