# Supplementary material for: An efficient coding theory for a dynamic trajectory predicts non-uniform allocation of entorhinal grid cells to modules
Source: PLoS Comput Biol. 2017 Jun 19;13(6):e1005597. doi: 10.1371/journal.pcbi.1005597 (PMC5495497; doi:10.1371/journal.pcbi.1005597)
Supplement: S1 Text — (PDF) [file pcbi.1005597.s001.pdf]

Supporting Information: An efficient coding theory for a dynamic trajectory predicts non-uniform allocation of entorhinal grid cells to modules*

Noga Weiss Mosheiff,[1] Haggai Agmon,[2] Avraham Moriel,[1] and Yoram Burak[1,2]

[1] *Racah Institute of Physics, The Hebrew University of Jerusalem*

[2] *Edmond and Lily Safra Center for Brain Sciences, The Hebrew University of Jerusalem*

CONTENTS

List of Figures 1

I. Optimal readout 2

II. Simplified readout 4

A. Mean squared error due to the random walk statistics 5

B. Mean square error due to the spiking variability 7

C. Mean square error using an optimised rectangular kernel 8

D. Mean square error using an optimised exponential kernel 9

III. Efficient Nested Code 10

A. Optimized code for a random walk statistic 11

B. Generalization for other statistics of motion 12

IV. Fisher information rate for Gaussian receptive fields 15

LIST OF FIGURES

S1 9

S2 10

S3 13

S4 17

---

* Submitted to PLoS Computational Biology

I. OPTIMAL READOUT

In this section we evaluate the local mean square error (MSE) of an optimal estimator for the instantaneous value of a dynamic random variable, based on all spikes emitted up to the decoding time. We assume that the variable follows the statistics of a simple random walk in two dimensions.

Our main interest is in a population of grid cells which all belong to the same module. In this case, when the rate of spikes is sufficiently high, the posterior consists of a periodic array of Gaussians. To simplify the exposition, we consider first the mean square error for receptive fields that are unimodal and aperiodic. Our derived expression for the MSE for this scenario applies to the *local* MSE in the case of periodic receptive fields.

We thus consider a two dimensional position $\vec{x}$, and a population of neurons that respond to the position with unimodal, translationally invariant receptive fields. Neuron i fires as an inhomogeneous Poisson process with rate

$$\Lambda_i(t) = f\left[\vec{x}(t) - \vec{x}_i\right] \tag{S1}$$

where $f(\vec{x})$ is the shape of the tuning curve and $\vec{x}_i$ is the center of the receptive field of neuron i. We assume that f decays to zero for large $|\vec{x}|$ and that $\vec{x}_i$ sample uniformly and densely the range of possible positions $\vec{x}$.

Due to the Markovian nature of the trajectory and the spiking statistics, it is sufficient for an optimal (maximum likelihood) decoder of position to track the posterior probability distribution over the current position of the animal, based on all the spikes observed up to time t. We denote this quantity as $p(\vec{x}, t)$. Within a short time interval dt, this quantity is updated as follows:

$$p\left(\vec{x}; t + \mathrm{d}t\right) = \frac{1}{Z}\left[\int \mathrm{d}\vec{x}' p_{\mathrm{D}}\left(\vec{x}|\vec{x}'\right) p\left(\vec{x}'; t\right)\right] p_{\mathrm{spikes}}(\vec{x}; t) \tag{S2}$$

where $p_{\mathrm{D}}(\vec{x}|\vec{x}')$ is the probability for the random walk to reach $\vec{x}$ at time $t + dt$ from position $\vec{x}'$ at time t. For a random walk in two dimensions

$$p_{\mathrm{D}}(\vec{x}|\vec{x}') = \frac{1}{4\pi D \mathrm{d}t} \exp\left(-\frac{|\vec{x} - \vec{x}'|^2}{4D\mathrm{d}t}\right). \tag{S3}$$

The last term in Eq. (S2) represents the likelihood of the spikes observed within the short time interval, given the position $\vec{x}$: let us denote by $r_i(t)$ the number of spikes emitted by neuron i between time t and time $t + \mathrm{d}t$. Then,

$$p_{\mathrm{spikes}}(\vec{x}; t) = \prod_i \mathrm{e}^{-f_i(\vec{x} - \vec{x}_i)dt} \frac{\left[f(\vec{x} - \vec{x}_i)dt\right]^{r_i}}{r_i!}. \tag{S4}$$

Finally, Z is a normalisation constant. Formally, the optimal decoder is obtained from the above decoder as its limiting form when $\mathrm{d}t \to 0$. We refer to such a decoder as a Bayesian decoder, since it optimally takes into account the prior over the statistics of the trajectory (a random walk with diffusion coefficient D).

We next consider the MSE of the maximum likelihood estimator

$$\hat{\vec{x}}_{\mathrm{ML}} = \mathrm{argmax}_{\vec{x}} p(\vec{x};t) \tag{S5}$$

This is the maximum likelihood estimate for $\vec{x}(t)$ based on all the spikes observed up to time t. A related quantity was treated in (Burak and Fiete, 2012): the mean square error of an optimal estimate for $\vec{x}(t)$ based on all spikes observed *after* time t. This latter problem, and the problem considered here are equivalent due to a symmetry of the random walk statistics with respect to time inversion. Hence we do not repeat the derivation here but cite the result: For motion in one dimension,

$$\Delta^2_{\mathrm{ML,1d}} \equiv \left\langle \left[\hat{x}_{\mathrm{ML}}(t) - x(t)\right]^2 \right\rangle = \sqrt{\frac{2D}{J}} \tag{S6}$$

where J is the Fisher information rate of the spiking neurons [21,22]

$$J = \sum_i \frac{\left[f'(\vec{x} - \vec{x}_i)\right]^2}{f(\vec{x} - \vec{x}_i)}\,, \tag{S7}$$

This quantity is independent of x due to the assumption of dense, translationally invariant receptive fields.

The result cited above is precise when J is sufficiently large (or the diffusion coefficient sufficiently small), that it is possible to choose a time interval $\mathrm{d}t$ for sequential update of the decoder's estimate, which obeys the following two requirements: First, an optimal estimator for a static position, based on spikes observed within an interval $\mathrm{d}t$, achieves the Cramér-Rao bound, $1/(J\mathrm{d}t)$. Second, the variance of diffusion within an interval $\mathrm{d}t$, $2D\mathrm{d}t$, is small compared to $1/(J\mathrm{d}t)$ and compared to the tuning curve width.

In two dimensions, if the tuning curve is isotropic, and under the above conditions, the posterior over position at any time is Gaussian, and it is possible to separate the decoding procedure to independent computations in each one of the two spatial directions. Thus, the MSE in each direction is given by Eq. (S6), where J is the Fisher information rate in a single direction. Altogether, we obtain

$$\Delta^2_{\mathrm{ML}} \equiv \left\langle \left[\hat{x}_{\mathrm{ML}}(t) - \vec{x}(t)\right]^2 \right\rangle = 2\sqrt{\frac{2D}{J}}\,. \tag{S8}$$

II. SIMPLIFIED READOUT

As in the previous section, we assume a population of neurons that span uniformly and densely the range of positions with translationally invariant, unimodal receptive fields. We assume that the Fisher information rate of spikes is high: more precise requirements are described within the derivation.

When the variable $\vec{x}$ is static, it is straightforward to express the likelihood of spike counts r_i, observed within a temporal window of duration Δt, as a function of the position $\vec{x}$:

$$\begin{aligned} L = \log p\left(\{r_i\}\left|\vec{x}\right.\right) &= \sum_i f\left(\vec{x}-\vec{x}_i\right)\Delta t + \sum_i r_i \ln\left\{f\left(\vec{x}-\vec{x}_i\right)\Delta t\right\} - \sum_i r_i! \\ &= c + \sum_i r_i \ln f\left(\vec{x}-\vec{x}_i\right)\,, \end{aligned} \tag{S9}$$

where c is a constant that does not depend on $\vec{x}$. The term $\sum_i f\left(\vec{x}-\vec{x}_i\right)$ contributes only to this constant because of the assumption of dense, translationally invariant receptive fields with uniform distribution. A maximum likelihood estimator for $\vec{x}$ (assuming a uniform prior) will choose

$$\hat{\vec{x}}\left(\{r_i\}\right) = \text{argmax}_{\vec{x}} \sum_i r_i \ln f\left(\vec{x}-\vec{x}_i\right)\,. \tag{S10}$$

Thus, the maximum likelihood estimate for a static variable $\vec{x}$ is linear in the spike counts.

We next wish to evaluate the accuracy in which such a decoder estimates $\vec{x}(t)$ when the variable $\vec{x}(t)$ is not static, but follows the statistics of a simple random walk with diffusion coefficient D. Without much added effort, we can consider a generalised form of this decoder, in which the spikes from the recent history are weighted with a temporal kernel $h(t)$. Thus, we generalise r_i to:

$$r_i(t) \equiv \int_{-\infty}^{t} \mathrm{d}t' h(t-t')\xi_i(t')\,. \tag{S11}$$

Here $\xi_i(t)$ is a series of delta functions that represents the spikes of neuron i. The counting of spikes within a temporal window of duration Δt is recovered under this definition by setting $h(t)$ equal to

$$h_{\text{rect}}(t) = \begin{cases} 1 & ; \quad t < \Delta t \\ 0 & ; \quad t > \Delta t \end{cases} \tag{S12}$$

We will be particularly interested in the exponential kernel

$$h_{\text{exp}}(t) = \exp\left(-\frac{t}{\tau}\right) \tag{S13}$$

where τ is positive.

Our goal is to evaluate the mean square error of the estimator defined by Eqs. (S10) and (S11):

$$V \equiv \left\langle |\hat{x}(t) - \vec{x}(t)|^2 \right\rangle \tag{S14}$$

where the averaging is both on the spiking variability of the encoding neurons and on the random walk statistics of $\vec{x}(t)$. We approximate V by the leading order contributions to an expansion in the two parameters that control the variablity: D and J^{-1}. To evaluate the leading order terms of this expansion, we calculate the contribution V_{traj} coming from the variability in the trajectory $\vec{x}(t)$, calculated while ignoring the spiking variability (thus, in the limit of vanishing J^{-1}); and the contribution V_{spikes} coming from the spiking variability, evaluated in the limit of vanishing D, *i.e.*, as if the position is static and equal to $\vec{x}(t)$. The leading order expression for V is simply the sum of V_{traj} and V_{spikes}. Corrections to this expression come from higher order terms. The approximation becomes precise when both contributions to the mean square error are small. Thus, the parameter regime in which the assumption is valid can be inferred later, once we obtain expressions for the two contributions.

A. Mean squared error due to the random walk statistics

Here we evaluate the mean square error coming from the dynamics of $\vec{x}$, while ignoring the spiking variability. Thus, we replace $r_i(t)$ by their mean over the spiking statistics:

$$\langle r_i \rangle_{\text{spikes}} = \int_{-\infty}^{t} \mathrm{d}t' h(t-t') f\left[\vec{x}(t') - \vec{x}_i\right] \,. \tag{S15}$$

With this replacement, $\hat{\vec{x}}$ [Eq. (S10)] becomes

$$\hat{\vec{x}} = \text{argmax}_{\vec{x}_0} \sum_i \int_{-\infty}^{t} \mathrm{d}t' h(t-t') f\left[\vec{x}(t') - \vec{x}_i\right] \ln f\left[\vec{x}_0 - \vec{x}_i\right] \,. \tag{S16}$$

Next, we derive a simpler expression for $\hat{\vec{x}}$, in terms of the recent history of motion. Since $\hat{\vec{x}}$ maximises the sum over i in this expression, we demand that the derivatives with respect to both components of $\vec{x}_0$ vanish:

$$\sum_i \int_{-\infty}^{t} \mathrm{d}t' h(t-t') f\left[\vec{x}(t') - \vec{x}_i\right] \frac{\partial_{x_j} f\left[\hat{x} - \vec{x}_i\right]}{f\left[\hat{x} - \vec{x}_i\right]} = 0 \tag{S17}$$

where $j = 1, 2$. To derive a simpler expression for $\hat{\vec{x}}$ we assume that $f(t)$ spans a sufficiently short time interval such that throughout this interval $f\left[\vec{x}(t') - \vec{x}_i\right]$ can be approximated by its taylor expansion around $\vec{x}(t) - \vec{x}_i$:

$$f\left[\vec{x}(t') - \vec{x}_i\right] \simeq f\left[\vec{x}(t) - \vec{x}_i\right] + \partial_{x_1} f\left[\vec{x}(t) - \vec{x}_i\right] \Delta x_1(t', t) + \partial_{x_2} f\left[\vec{x}(t) - \vec{x}_i\right] \Delta x_2(t', t) \,, \tag{S18}$$

where

$$\Delta\vec{x}(t',t) \equiv \vec{x}(t') - \vec{x}(t)\,. \tag{S19}$$

We first note that if we truncate this expansion at the leading term and use this approximation in Eq. (S17), we find that the equation is obeyed for $\hat{\vec{x}} = \vec{x}(t)$: Indeed, under the assumption of dense and uniform receptive fields, plugging in Eq. (S17) $f\left[\vec{x}(t) - \vec{x}_i\right]$ instead of $f\left[\vec{x}(t') - \vec{x}_i\right]$ and $\vec{x}(t)$ instead of $\hat{\vec{x}}$ yields

$$\int_{-\infty}^{t} \mathrm{d}t' h(t-t') \sum_i \partial_{x_j} f\left[\vec{x}(t) - \vec{x}_i\right] \propto \int_{-\infty}^{\infty} \partial_{x_j} f(\vec{x}) \mathrm{d}\vec{x} = 0\,. \tag{S20}$$

To obtain the deviation of $\hat{\vec{x}}$ from $\vec{x}(t)$ we need to take into account the terms in Eq. (S18) that are linear in $\Delta\vec{x}$. We first write:

$$\hat{\vec{x}} = \vec{x}(t) + \Delta\hat{x}\,. \tag{S21}$$

Then, to leading order in $\Delta\hat{x}$ and in $\Delta\vec{x}(t',t)$ we get from Eqs. (S17), (S18), and (S21):

$$\begin{aligned}
0 = &\sum_i \int_{-\infty}^{t} \mathrm{d}t' h(t-t') \left\{ \partial_{x_1}^2 f\left[\vec{x}(t) - \vec{x}_i\right] - \frac{\left(\partial_{x_1} f\left[\vec{x}(t) - \vec{x}_i\right]\right)^2}{f\left[\vec{x}(t) - \vec{x}_i\right]} \right\} \Delta\hat{x}_1 \\
&+ \sum_i \int_{-\infty}^{t} \mathrm{d}t' h(t-t') \left\{ \partial_{x_1}\partial_{x_2} f\left[\vec{x}(t) - \vec{x}_i\right] - \frac{\partial_{x_1} f\left[\vec{x}(t) - \vec{x}_i\right] \partial_{x_2} f\left[\vec{x}(t) - \vec{x}_i\right]}{f\left[\vec{x}(t) - \vec{x}_i\right]} \right\} \Delta\hat{x}_2 \\
&+ \sum_i \int_{-\infty}^{t} \mathrm{d}t' h(t-t') \frac{\left(\partial_{x_1} f\left[\vec{x}(t) - \vec{x}_i\right]\right)^2}{f\left[\vec{x}(t) - \vec{x}_i\right]} \Delta x_1(t',t) \\
&+ \sum_i \int_{-\infty}^{t} \mathrm{d}t' h(t-t') \frac{\partial_{x_1} f\left[\vec{x}(t) - \vec{x}_i\right] \partial_{x_2} f\left[\vec{x}(t) - \vec{x}_i\right]}{f\left[\vec{x}(t) - \vec{x}_i\right]} \Delta x_2(t',t)
\end{aligned} \tag{S22}$$

where without loss of generality we chose $j = 1$ in Eq. (S17). As in Eq. (S20), it is easy to see that the terms involving second derivatives vanish. A few simple additional steps lead to the equation

$$\int_{-\infty}^{t} h(t-t') \Delta x_j(t',t) \mathrm{d}t' = \int_{-\infty}^{t} h(t-t') \mathrm{d}t' \Delta\hat{x}_j \tag{S23}$$

from which we see that

$$\hat{\vec{x}} = \frac{1}{H} \int h(t-t') \vec{x}(t') \mathrm{d}t' \tag{S24}$$

where

$$H \equiv \int_{-\infty}^{t} h(t-t') \mathrm{d}t'\,. \tag{S25}$$

Thus, we reach a simple and intuitive result, that $\hat{\vec{x}}$ is given by a temporal average of $\vec{x}$ in the recent history, weighted by the kernel h.

Using the expression (S24) which relates $\hat{\vec{x}}$ to the trajectory, and assuming stationarity, it is straightforward to evaluate the mean square error generated by the stochastic dynamics of $\vec{x}$:

$$\begin{aligned} V_{\rm traj} \equiv \left\langle \left| \hat{\vec{x}} - \vec{x}(t) \right|^2 \right\rangle &= \frac{1}{H^2} \int_{-\infty}^{t} {\rm d}t' \int_{-\infty}^{t} {\rm d}t'' h(t-t')h(t-t'') \left\langle \left[\vec{x}(t') - \vec{x}(t)\right] \cdot \left[\vec{x}(t'') - \vec{x}(t)\right] \right\rangle \\ &= \frac{1}{H^2} \int_{-\infty}^{t} {\rm d}t' \int_{-\infty}^{t'} {\rm d}t'' h(t-t')h(t-t'') \cdot \\ &\quad \cdot \left[\left\langle \left|\Delta\vec{x}(t',t)\right|^2 \right\rangle + \left\langle \left|\Delta\vec{x}(t'',t)\right|^2 \right\rangle - \left\langle \left|\Delta\vec{x}(t',t'')\right|^2 \right\rangle \right] \end{aligned} \tag{S26}$$

For $t' < t$ we have:

$$\left\langle \left|\Delta\vec{x}(t',t)\right|^2 \right\rangle = 4D(t-t') \,. \tag{S27}$$

Therefore, for $t'' < t' < t$

$$V_{\rm traj} = \left\langle \left| \hat{\vec{x}} - \vec{x}(t) \right|^2 \right\rangle_{\rm traj} = \frac{8D}{H^2} \int_{-\infty}^{t} {\rm d}t' \int_{-\infty}^{t'} {\rm d}t'' \left(t-t'\right) h(t-t')h(t-t'') \,. \tag{S28}$$

For the rectangular kernel, Eq. (S12), we get

$$V_{\rm traj,rect} = \frac{4}{3} D\Delta t \,, \tag{S29}$$

and for the exponential kernel, Eq. (S13) we get

$$V_{\rm traj,exp} = 2D\tau \,. \tag{S30}$$

B. Mean square error due to the spiking variability

We next wish to evaluate the mean square error due to the spiking variability, while assuming that the position $\vec{x}$ is static. For the rectangular kernel (S12) the answer is immediate: $\hat{\vec{x}}$ of Eq. (S10) is the maximum likelihood estimate for $\vec{x}$ given the spikes emitted within an interval of duration Δt. Therefore, for a rectangular kernel

$$V_{\rm spikes,rect} = \frac{2}{J\Delta t} \tag{S31}$$

where J is the Fisher information rate of the Poisson spiking neurons, and the factor 2 comes from the two dimensions (provided that $J\Delta t$ is sufficiently large, such that the Cramér Rao bound is saturated). More generally, we observe that in Eq. (S10), $\sum_i r_i \ln f\left(\vec{x} - \vec{x}_i\right)$ has a normal distribution when the sum involves many independent contributions (either many neurons or many spikes). To understand the effect of noise variability it is then sufficient to evaluate the first and second moments of this expression. It is more convenient to consider directly the variables r_i, which are

statistically independent (although these don't necessarily need to be normally distributed, since many of them are summed up to generate the estimate). Their mean is equal to

$$\langle r_i \rangle = H f(\vec{x} - \vec{x}_i)\,, \tag{S32}$$

and their variance is equal to

$$(\Delta r_i)^2 = \left[\int_0^\infty h^2(t)\mathrm{d}t\right] f(\vec{x} - \vec{x}_i)\,, \tag{S33}$$

where we made use of the Poisson statistics of the spikes.

For the rectangular window (S12), for which $h^2 = h$, the variance is equal to the mean, as expected since r_i is Poisson distributed. The signal to noise ratio is equal to

$$\frac{\langle r_i \rangle^2}{(\Delta r_i)^2} = f(\vec{x} - \vec{x}_i)\Delta t\,. \tag{S34}$$

For a general choice of h, the signal to noise ratio is equal to

$$\frac{\langle r_i \rangle^2}{(\Delta r_i)^2} = f(\vec{x} - \vec{x}_i) \cdot \frac{H^2}{\int_0^\infty h^2(t)\mathrm{d}t} \tag{S35}$$

Hence, the r_i have the same signal to noise ratio as the spike count from an interval of duration

$$\Delta t_{\text{effective}} = \frac{H^2}{\int_0^\infty h^2(t)\mathrm{d}t}\,. \tag{S36}$$

We conclude that the variance of the estimator is given by

$$V_{\text{spikes}} = \frac{\int_0^\infty h^2(t)\mathrm{d}t}{H^2} \cdot \frac{2}{J}\,. \tag{S37}$$

For the exponential kernel in particular we find

$$V_{\text{spikes,exp}} = \frac{1}{J\tau}\,. \tag{S38}$$

C. Mean square error using an optimised rectangular kernel

We can now put these results together, and express the mean square error of the estimator with a rectangular kernel using Eqs.(S29) and (S31),

$$V_{\text{rect}} = \frac{2}{J\Delta t} + \frac{4D\Delta t}{3}\,. \tag{S39}$$

An example is plotted in Fig. S1, where it is compared with the MSE evaluated in numerical simulations, showing excellent agreement. The minimum of this expression is achieved by choosing

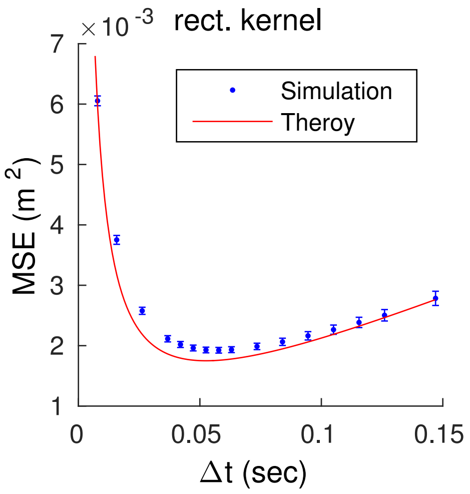

FIG. S1. **Local MSE of a kernel decoder with a rectangular kernel.** The local MSE for estimate of position from spikes in a single module, using the rectangular kernel. The blue dots are simulations of the MSEs where each dot is an average over 200 realizations and for each realization the MSE was computed by averaging over time. The margins of error are 1.96 standard deviations of the MSEs divided by the square root of the number of realizations. For each realization we simulate 600 neurons with Gaussian receptive fields where $r_{\max} = 10Hz$, and $\lambda = 1m$. The red line is the theoretical prediction from Eq. (S39).

$\Delta t = \Delta t^*$,

$$\Delta t^* = \sqrt{\frac{3}{2DJ}} \tag{S40}$$

which yields a MSE of readout:

$$\Delta^2_{\text{rect}} \equiv V_{\text{rect}}\left(\Delta t^*\right) = \sqrt{\frac{16}{3}}\sqrt{\frac{2D}{J}}\,. \tag{S41}$$

This mean square error is slightly larger than that of an optimal Bayesian estimator, Eq. (S8). At the optimal Δt, $V_{\text{spikes,rect}}$ and $V_{\text{traj,rect}}$ have identical magnitude. In order for the derivation to be valid, both should be small compared to the receptive field width. Another derivation of this result, under a somewhat different context, is given in [24].

D. Mean square error using an optimised exponential kernel

For the exponential kernel, we use Eqs. (S30) and (S38). We obtain

$$V_{\exp} = \frac{1}{J\tau} + 2D\tau\,. \tag{S42}$$

An example, which demonstrates excellent agreement between simulations and this result, is plotted in Fig. S2. The minimum of $V_{\exp}$ is achieved by choosing $\tau = \tau^*$,

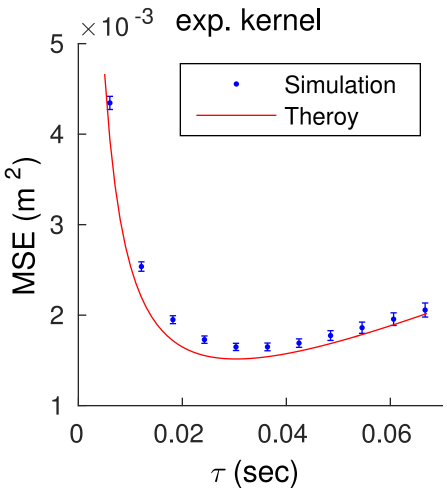

FIG. S2. **Local MSE of a kernel decoder with an exponential kernel.** The MSE of the kernel estimator based on spikes from a single module, using an exponential kernel, shown as a function of the integration time τ. The blue dots are evaluations of the MSE from simulations, and the red line is the theoretical prediction, Eq. (S42). The parameters are the same as in Fig. S1. Note that the minimum of V_{exp} is lower that the minimum of V_{rect} (Fig. S1).

$$\tau^* = \sqrt{\frac{1}{2DJ}}, \tag{S43}$$

which yields a MSE of readout:

$$\Delta^2_{\mathrm{exp}} \equiv V_{\mathrm{exp}}\left(\tau^*\right) = 2\sqrt{\frac{2D}{J}}. \tag{S44}$$

Thus, the estimator achieves the same mean square error as that of the optimal Bayesian estimator (in the limit of sufficiently large Fisher information). This is a remarkable result, since the computation performed by the kernel estimator can be easily implemented by neural circuitry, and is significantly simpler than required in a direct implementation of the optimal Bayesian estimator.

III. EFFICIENT NESTED CODE

We consider a nested code, in which position can be read out sequentially starting from the module with the largest spacing, progressing sequentially to modules with smaller grid spacings. Our goal is to minimize the local RMSE of the module with the smallest grid spacing Δ_m, where m is the number of modules, and $\lambda_1 > \lambda_2 > ... > \lambda_m$, while constraining the total number of neurons N, and the largest grid spacing λ_1.

A. Optimized code for a random walk statistic

Assuming that the animal's trajectory follows the statistics of a simple random walk, we obtain from Eqs. (2),(3) and (5) the relation:

$$\Delta_m^2 = 2\sqrt{\tfrac{2D}{J_m}} = 2\sqrt{\tfrac{2D}{\alpha n_m}}\lambda_m = \left(\tfrac{8D}{\alpha n_m}\right)^{\frac{1}{2}} \frac{1}{\beta}\Delta_{m-1} \tag{S45}$$

An expression for Δ_m is obtained by iterating this relation:

$$\Delta_m^2 = A_m \cdot \prod_{i=1}^{m} \left(\tfrac{1}{n_i}\right)^{\left(\frac{1}{2}\right)^{m+1-i}} , \tag{S46}$$

where $A_m = \lambda_1^{\left(\frac{1}{2}\right)^{m-1}} \left(\frac{1}{\beta}\right)^{2-\left(\frac{1}{2}\right)^{m-2}} \left(\frac{8D}{\alpha}\right)^{1-\left(\frac{1}{2}\right)^{m}}$.

To find the optimal n_i, we consider the Lagrangian:

$$\mathcal{L} = \log\left(\Delta_m\right) + \gamma \left(\sum_{i=1}^{m} \frac{n_i}{N} - 1\right) . \tag{S47}$$

The second term of $\mathcal{L}$ reflects the constraint over N, where γ is a Lagrange multiplier. The minimization of Δ_m is obtained from the derivatives:

$$\frac{\partial \mathcal{L}}{\partial n_i} = 0 \,, \tag{S48}$$

$$\frac{\partial \mathcal{L}}{\partial \gamma} = 0 \,, \tag{S49}$$

which result in the allocation:

$$n_i = \frac{\left(\frac{1}{2}\right)^{m+1-i}}{1-\left(\frac{1}{2}\right)^{m}} \cdot N \,, \tag{S50}$$

and lead to Eq. (6), since

$$\frac{n_{i+1}}{n_i} = \frac{\left(\frac{1}{2}\right)^{m-i}}{\left(\frac{1}{2}\right)^{m+1-i}} = 2 \,. \tag{S51}$$

Note that this result does not depend on any of the parameters of the problem (such as β, N, m, D or λ_1). In similarity to the derivation of Eq. (S46), the expression for MSE from the i'th module is:

$$\Delta_i^2 = A_i \prod_{j=1}^{i} \left(\tfrac{1}{n_j}\right)^{\left(\frac{1}{2}\right)^{i+1-j}} . \tag{S52}$$

By substitution Eq. (S50) in this expression we obtain, after some algebra, a prediction for the ratio between grid spacing of following modules:

$$\frac{\lambda_i}{\lambda_{i+1}} = \frac{\Delta_{i-1}}{\Delta_i} = \sqrt{2} \cdot C^{\left(\frac{1}{2}\right)^i}, \tag{S53}$$

where

$$C = \lambda_1 \sqrt{\frac{\alpha N \beta^4}{8D\left(1\text{-}\left(\frac{1}{2}\right)^m\right)}} \left(\frac{1}{2}\right)^{\frac{m+2}{2}} . \tag{S54}$$

Thus, this ratio approaches, for the modules with small spacing, the limit $\sqrt{2}$, as can be seen in Fig. 2. Eq. (S53) also predicts that the ratios between grid spacing is a monotonic sequence. This sequence might increase or decrease, depending of the value of C. If $C > 1$ the grid spacing ratio decreases monotonically with the decreasing spacing (as in Fig. 2). However, if $C < 1$ this ratio increases. With the parameters of Fig. 2 in the case of $N = 10^4$, $C \simeq 1.05$. Therefore we are not capable to predict whether the ratio decreases or increases. In the particular case of $C = 1$ the ratios between grid spacing would be constant. However, this case requires fine tuning of the parameters. Hence, we believe this is not the common case.

The parameter β [Eq. (5)] was chosen in Figs. 2–4 as follows. This parameter should be set small enough to ensure that there are no global ambiguities, since the minimization of Δ_m affects only the local inference error. We applied the minimization procedure with various values of β to select n_i and λ_i, and then evaluated the MSE of the exponential kernel estimator. As expected, very small values of β led to large MSE, since β sets a limit on the degree of reduction in Δ from module to module. Large values of β also led to large MSE due to errors arising from global ambiguities (Fig. S3 and Fig. 2A). We chose in the simulations $\beta = 0.1$, as this value provides an MSE close to minimal (we did not attempt to find the precise optimal value of β).

B. Generalization for other statistics of motion

We next generalize our optimized code for statistics of motion besides the simple random walk. We consider all cases in which the variance of translation in space follows a power law as a function of the elapsed time. Thus, we replace equation (S27) by Eq. (8). For a simple random walk in two dimensions $g = 4D$ and $\epsilon = 1$, while for a trajectory of constant velocity v, $g = v^2$ and $\epsilon = 2$. In order to deal with such a motion, we focus on the simplified readout scheme of spikes based on the exponential temporal kernel. Thus, Eq. (3) is replaced by an expression which represents the local MSE of readout from a single module, according to the modified statistics of motion. In our

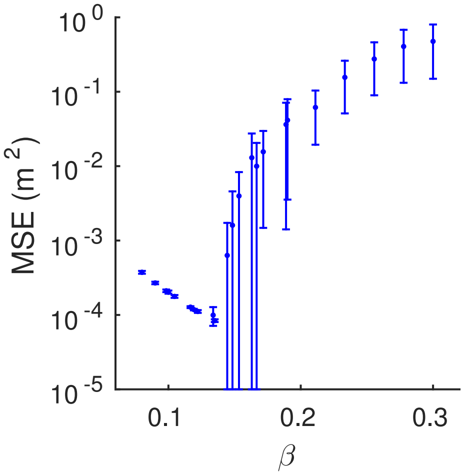

FIG. S3. **Dependence of the MSE on parameter β.** MSE of the exponential kernel decoder, estimating position based on spikes from all modules, for different values of the parameter β. The blue dots are simulations of the MSEs where each dot represents an average over time and over 20 realizations, each lasting $\sim$ 700ms. The margins of error are 1.96 standard deviations of the MSEs divided by square root of the number of realizations. The parameters are the same as in Fig. 2 with $N = 10^4$. It can be seen that the MSE has a minimum for some $0.1 < \beta < 0.2$. The sharp increase in the MSE for larger values of β is the result of ambiguities in the inference of the animal's position, which occasionally result in a very large displacement between the estimate and the true position. When β is larger than the minimum but close to it, these events occur rarely, but generate a large contribution to the MSE. The large error bars are due to the sparseness of these events.

notation, Eq. (8) becomes

$$\left\langle \left|\Delta\vec{x}(t',t)\right|^2 \right\rangle = g\left(\left|t'-t\right|\right)^{\epsilon} . \tag{S55}$$

By substituting Eq. (S55) and the exponential kernel Eq. (S13) in Eq. (S26) we obtain:

$$V_{\text{traj,exp}} = \frac{g\tau^{\epsilon}}{2}\Gamma\left(\epsilon+1\right) , \tag{S56}$$

where Γ is the gamma function. Hence, Eq. (S42) is replaced by

$$V_{\text{exp}} = \frac{1}{J\tau} + \frac{g\tau^{\epsilon}}{2}\Gamma\left(\epsilon+1\right) . \tag{S57}$$

The minimum of this expression is achieved by choosing

$$\tau^* = \left(\frac{2}{Jg\epsilon\Gamma\left(\epsilon+1\right)}\right)^{\frac{1}{\epsilon+1}} , \tag{S58}$$

which yields a MSE of readout:

$$\Delta^2_{\text{exp}} \equiv V_{\text{exp}}\left(\tau^*\right) = \left[\frac{g\epsilon}{2}\Gamma\left(\epsilon+1\right)\right]^{\frac{1}{\epsilon+1}} \left(1+\frac{1}{\epsilon}\right)\left(\frac{1}{J}\right)^{\frac{\epsilon}{\epsilon+1}} = \frac{M}{J^k} , \tag{S59}$$

where

$$k \equiv \frac{\epsilon}{\epsilon + 1} \tag{S60}$$

and

$$M \equiv \left[\frac{g\epsilon}{2} \Gamma\left(\epsilon + 1\right) \right]^{\frac{1}{\epsilon+1}} \left(1 + \frac{1}{\epsilon}\right) . \tag{S61}$$

The constants M and k depend only on ϵ and g, which are deduced directly from the variance of motion [Eq. (8)]. Note that the scaling with J and g in Eqs. (S58)-(S59) can be deduced based on dimensional analysis. Therefore, this scaling is likely not specific only to the class of decoders considered here.

Now, similarly to the case of random walk motion, we can derive an expression for Δ_m^2, while replacing Eq. (3) with Eq. (S59). This leads to the result,

$$\Delta_m^2 = \tilde{A}_m \prod_{i=1}^{m} \left(\tfrac{1}{n_i}\right)^{k^{m+1-i}} , \tag{S62}$$

where

$$\tilde{A}_m = \frac{M \lambda_1^{2k^m}}{\alpha^{k^m}} \left(\frac{M}{\alpha\beta^2}\right)^{\frac{k^m - k}{k-1}} . \tag{S63}$$

By the same minimization process that was used for the case of random walk motion, we obtain the allocation:

$$n_i = \frac{1-k}{1-k^m} k^{m-i} N . \tag{S64}$$

Hence,

$$\frac{n_{i+1}}{n_i} = \frac{1}{k} = \frac{\epsilon + 1}{\epsilon} , \tag{S65}$$

as stated by Eq. (9). For a simple random walk we reproduce our former result ($n_{i+1}/n_i = 2$), whereas for motion at constant velocity $n_{i+1}/n_i = 1.5$. The ratio between successive grid spacings is

$$\frac{\lambda_i}{\lambda_{i+1}} = \left(\frac{1}{k}\right)^{\frac{k}{2(1-k)}} \tilde{C}^{k^i} , \tag{S66}$$

where

$$\tilde{C} = \lambda_1^{\frac{1-k}{k}} \beta^{\frac{1}{k}} \sqrt{\frac{\alpha N}{M^{\frac{1}{k}}} \frac{1-k}{1-k^m} k^{\left(m + \frac{k}{1-k}\right)}} . \tag{S67}$$

This ratio approaches, for the modules with small spacing (see Fig. 5):

$$\frac{\lambda_i}{\lambda_{i+1}} \to \left(\frac{1}{k}\right)^{\frac{k}{2(1-k)}} = \left(\frac{\epsilon+1}{\epsilon}\right)^{\frac{\epsilon}{2}} \tag{S68}$$

as stated by Eq. (10). This ratio varies monotonically between $\sqrt{2} \simeq 1.41$ to 1.5 in the interval $\epsilon = [1, 2]$. In addition to this result, Eq. (S66) predicts that the grid spacings form a monotonic sequence, in similarity to the case of random walk statistics (see Eq. (S53) and the subsequent discussion).

IV. FISHER INFORMATION RATE FOR GAUSSIAN RECEPTIVE FIELDS

Consider a uniform and dense cover of the plane by n independent Poisson grid cells from a single module, with Gaussian receptive fields:

$$f(\vec{x}) = r_{\mathrm{max}} \sum_{\alpha} \exp\left[-\frac{|\vec{x}-\vec{x}_{\alpha}|^2}{2\sigma^2}\right] \tag{S69}$$

where r_{max} is the neuron maximal firing rate and the sum is over all the discrete translations of the periodic lattice, $\{\vec{x}_{\alpha}\}$.

The Fisher information matrix has the form:

$$\overleftrightarrow{J} = \begin{pmatrix} J_{xx} & J_{xy} \\ J_{yx} & J_{yy} \end{pmatrix} \tag{S70}$$

where due to symmetry $J_{xx} = J_{yy} \equiv J$. For independent Poisson neurons [21,22]:

$$J = J_{xx} = \sum_{i=1}^{n} \frac{1}{f(\vec{x}-\vec{x}_i)} \left[\frac{\partial}{\partial x} f(\vec{x}-\vec{x}_i)\right]^2 \tag{S71}$$

where the receptive field centers $\vec{x}_i$ uniformly and densely span the unit cell of the periodic lattice. Using the assumption of uniform, dense sampling, we can replace the sum in Eq. (S71) by an integral over a unit cell. Furthermore, assuming that the width of each firing field σ is small compared to the periodicity of the lattice, only one firing field (at most) in the sum over α contributes to the Fisher information. We can include a single Gaussian in the integral and extend the limits of integration to infinity:

$$J = \frac{n}{A} \int_{-\infty}^{\infty} \mathrm{d}x \int_{-\infty}^{\infty} \mathrm{d}y \frac{r_{\mathrm{max}}}{\sigma^4} x^2 \exp\left(-\frac{x^2+y^2}{2\sigma^2}\right) = \frac{4\pi}{\sqrt{3}} \cdot \frac{n r_{\mathrm{max}}}{\lambda^2} \tag{S72}$$

where $A = (\sqrt{3}/2)\lambda^2$ is the area of the unit cell. For the non-diagonal elements we similarly obtain $J_{xy} = J_{yx} = 0$. Thus, we conclude that the fisher information matrix is:

$$\overleftrightarrow{J} = \begin{pmatrix} J & 0 \\ 0 & J \end{pmatrix} \tag{S73}$$

where

$$J = \frac{4\pi}{\sqrt{3}} \cdot \frac{n r_{\max}}{\lambda^2} \tag{S74}$$

We note that, as expected for encoding in two dimensions, the Fisher Information is independent of the choice of σ^2. This, however, is correct only as long as σ is small compared to λ, and large compared to the distance between neighboring receptive field centers.

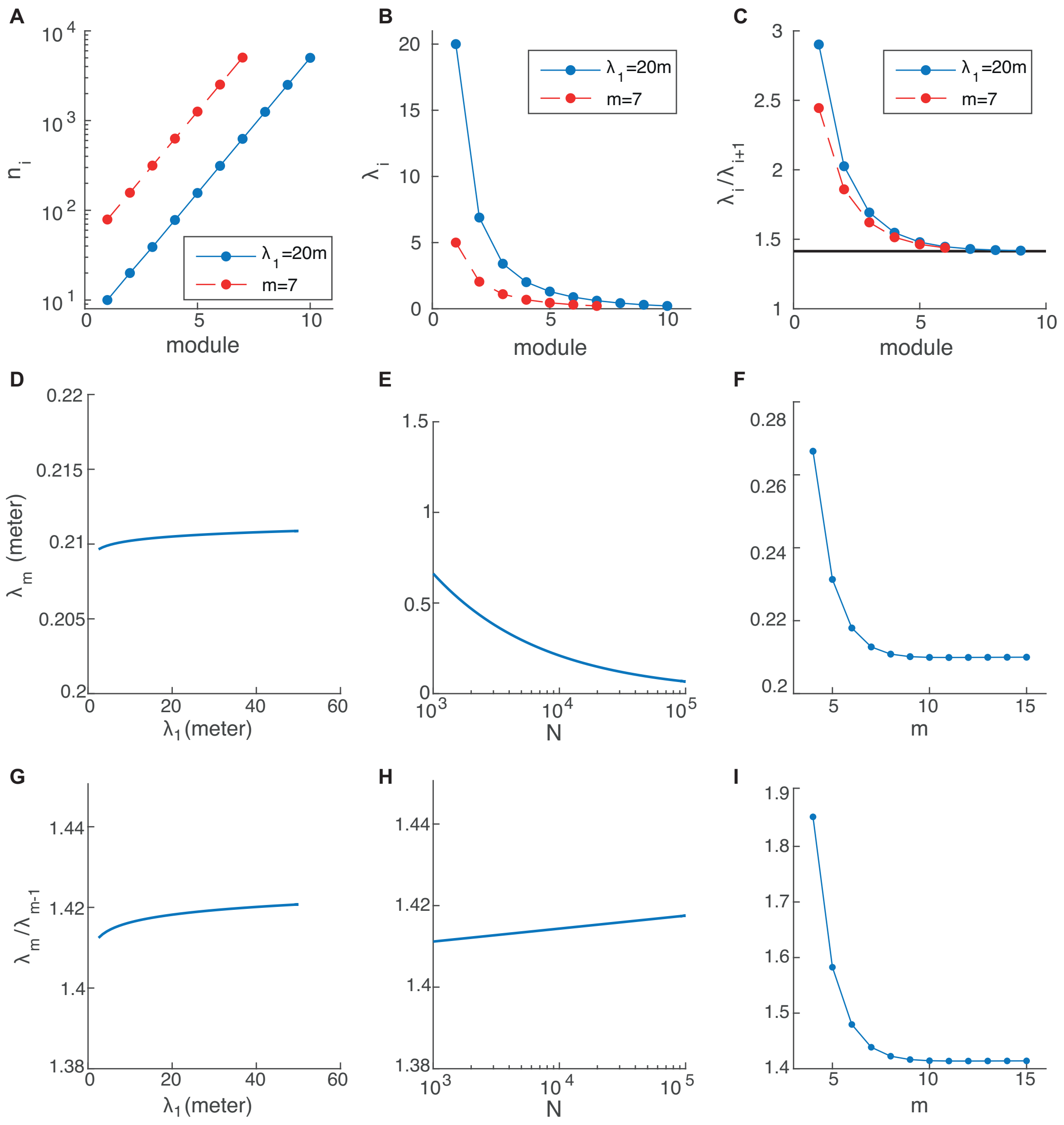

FIG. S4. **Results of optimization for different choices of the largest grid spacing, the number of modules and the total number of cells. A.-C.** Analytical results as shown in Fig. 2B-D, using the same parameters as in Fig. 2 for $N = 10^4$ except for a change in one parameter: $\lambda_1 = 20$m (blue) or the number of modules $m = 7$ (red). The detailed spacings depend on the specific parameters. However, the central properties discussed in the manuscript are unchanged (the constant ratio between module population sizes [Eq. (6)], and the approximately constant ratio between subsequent grid spacings, for small spacings. **D.-F.** The smallest grid spacing λ_m as a function of several parameters. **G.-I.** λ_m/λ_{m-1} as a function of the same parameters. The results are fairly insensitive to changes in λ_1 (**D,G**) or in m, for sufficiently large m (**F,I**). Changing the total number of cells does affect the smallest grid spacing λ_m (**E**).
